# Prenatal Exposure to Perfluorooctanoate and Risk of Overweight at 20 Years of Age: A Prospective Cohort Study

**DOI:** 10.1289/ehp.1104034

**Published:** 2012-02-03

**Authors:** Thorhallur I. Halldorsson, Dorte Rytter, Line Småstuen Haug, Bodil Hammer Bech, Inge Danielsen, Georg Becher, Tine Brink Henriksen, Sjurdur F. Olsen

**Affiliations:** 1 Center for Fetal Programming, Department of Epidemiology Research, Statens Serum Institute, Copenhagen, Denmark; 2 Faculty of Food Science and Nutrition, University of Iceland, Reykjavik, Iceland; 3 Unit for Nutrition Research, Landspitali University Hospital, Reykjavik, Iceland; 4 Department of Public Health, Section for Epidemiology, Aarhus University, Denmark; 5 Division of Environmental Medicine, Norwegian Institute of Public Health, Oslo, Norway; 6 Department of Analytical Chemistry, University of Oslo, Oslo, Norway; 7 Department of Paediatrics, Aarhus University Hospital, Skejby, Denmark; 8 Department of Nutrition, Harvard School of Public Health, Boston, Massachusetts, USA

**Keywords:** offspring obesity, overweight, perfluoroalkyl compounds, PFOA, pregnancy, prenatal exposure

## Abstract

Background: Perfluoroalkyl acids are persistent compounds used in various industrial -applications. Of these compounds, perfluorooctanoate (PFOA) is currently detected in humans worldwide. A recent study on low-dose developmental exposure to PFOA in mice reported increased weight and elevated biomarkers of adiposity in postpubertal female offspring.

Objective: We examined whether the findings of increased weight in postpubertal female mice could be replicated in humans.

Methods: A prospective cohort of 665 Danish pregnant women was recruited in 1988–1989 with offspring follow-up at 20 years. PFOA was measured in serum from gestational week 30. Offspring body mass index (BMI) and waist circumference were recorded at follow-up (*n* = 665), and biomarkers of adiposity were quantified in a subset (*n* = 422) of participants.

Results: After adjusting for covariates, including maternal pre-pregnancy BMI, smoking, education, and birth weight, *in utero* exposure to PFOA was positively associated with anthropometry at 20 years in female but not male offspring. Adjusted relative risks comparing the highest with lowest quartile (median: 5.8 vs. 2.3 ng/mL) of maternal PFOA concentration were 3.1 [95% confidence interval (CI): 1.4, 6.9] for overweight or obese (BMI ≥ 25 kg/m^2^) and 3.0 (95% CI: 1.3, 6.8) for waist circumference > 88 cm among female offspring. This corresponded to estimated increases of 1.6 kg/m^2^ (95% CI: 0.6, 2.6) and 4.3 cm (95% CI: 1.4, 7.3) in average BMI and waist circumference, respectively. In addition, maternal PFOA concentrations were positively associated with serum insulin and leptin levels and inversely associated with adiponectin levels in female offspring. Similar associations were observed for males, although point estimates were less precise because of fewer observations. Maternal perfluorooctane sulfonate (PFOS), perfluorooctane sulfonamide (PFOSA), and perfluorononanoate (PFNA) concentrations were not independently associated with offspring anthropometry at 20 years.

Conclusions: Our findings on the effects of low-dose developmental exposures to PFOA are in line with experimental results suggesting obesogenic effects in female offspring at 20 years of age.

Because of their repellent properties and low surface tension, fluorochemicals have been used extensively for more than half a century in various commercial applications, including carpets, textiles, personal care products, leveling and wetting agents and in food-contact materials (D’eon and Mabury 2011). Use and production of fluorochemicals has resulted in the environmental presence of perfluoroalkyl acids (PFAAs) (Giesy and Kannan 2002), which are highly persistent compounds consisting of a fully fluorinated carbon chain (C4–C14) with, for example, a terminal sulfonate or carboxylate functional group. The environmental presence of PFAAs may be a consequence of emissions during manufacturing, use, or disposal of products (direct sources) as well as transformation of other precursors to PFAAs (indirect sources) (Armitage et al. 2009; D’eon and Mabury 2011). The two PFAAs most widely detected in humans are the C8-backbone derivatives perfluorooctanoate (PFOA) and perfluoro-octane sulfonate (PFOS), with estimated elimination half-lives of around 4 and 5 years, respectively (Olsen et al. 2007).

After reports of global contamination, phaseout of C8-derivatives was initiated in the year 2000 by the major U.S. manufacturers (U.S. Environmental Protection Agency 2010). This has resulted in decreased human exposure compared with peak levels in Europe and the United States (Haug et al. 2009b; Olsen et al. 2011). However, recent findings from the Health and Nutrition Examination Survey (NHANES) indicate that PFOA levels in the United states remained relatively stable between 2003 and 2008, suggesting continued direct and indirect sources of human exposures to PFOA (Kato et al. 2011).

PFAAs accumulate primarily via binding to albumin in blood (D’eon et al. 2010; Jones et al. 2003), and they are readily transported across the placenta (Monroy et al. 2008). PFAAs are found in highest concentrations in the liver (Hundley et al. 2006; Maestri et al. 2006) and low-dose human exposures to PFOA and PFOS have been associated with modest increases in liver enzymes (Lin et al. 2010) and blood lipids (Nelson et al. 2010) in cross-sectional settings. The relevance of these findings is uncertain, however, because prospective studies have not been conducted. Findings from animal studies suggest that PFAAs may act as endocrine disruptors, affecting circulating estrogens levels through pathways such as aromatase induction in the liver (Liu et al. 1996) or through altering estrogen-expressive genes (Shi et al. 2009), although findings with respect to interactions with estrogen receptors have not been consistent (Benninghoff et al. 2011; Ishibashi et al. 2007).

Experimental studies have demonstrated that *in utero* exposures to certain endocrine disruptors, including xenoestrogens, may lead to permanent changes in metabolic pathways that regulate body weight (Newbold et al. 2008). A recent study using a CD-1 mouse model observed higher body weight and increased insulin and leptin levels in post-pubertal female offspring after low-dose *in utero* exposures to PFOA (Hines et al. 2009). The aim of this study was to explore these findings in environmentally exposed pregnant women with prospective offspring follow-up at 20 years of age.

## Methods

*The birth cohort.* Between April 1988 and January 1989, 965 women with singleton pregnancies were recruited for a birth cohort study in Aarhus, Denmark (Olsen et al. 1995). This was 80% of a consecutive sample of 1,212 women attending a midwife center in the city, covering a well-defined geographical area. A face-to-face interview was conducted at a routine midwife visit in gestational week 30, covering medical history, anthropometry, diet, lifestyle, and socioeconomic factors. A blood sample was also collected, and it was immediately separated into serum, plasma, and erythrocytes and frozen at –20°C. Further information on maternal health and birth outcomes were extracted from hospital records and the Danish Medical Birth Registry.

*Offspring follow-up.* In 2008–2009, study mothers and offspring were contacted, and offspring were asked to fill out a Web-based questionnaire concerning their current health, lifestyle, and dietary habits and their current height and weight. In addition, a tape measure was mailed to all potential participants with instructions on how to measure their waist circumference. Offspring were also invited to participate in a 75–100-min clinical examination, which included standardized anthropometric measures and collection of a fasting blood sample that was immediately centrifuged and frozen at –80°C. The study was approved by the Danish Data Protection Agency and the Danish Council of Ethics (reference no. 20070157), and all participants gave written consent prior to inclusion into the study.

*Mother–offspring pairs available for analysis.* A total of 915 offspring of the 965 women with singleton pregnancies recruited in 1988–1999 were located and contacted in 2008–2009, and 692 of the offspring agreed to participate in the follow-up study. Missing values on offspring anthropometry (*n* = 4) and maternal blood samples (*n* = 23) left 665 mother–-offspring pairs, or 69% of the original cohort, available for analyses. Of the 665 offspring included in this study, 423 (44% of the original cohort) attended the clinical examination, and 242 completed the Web-based questionnaire only.

*Maternal PFAA samples.* Serum samples from gestational week 30 were measured at the Department of Analytical Chemistry at the Norwegian Institute of Public Health in Oslo. The analytical procedure and sources of chemicals used have been described in detail elsewhere (Haug et al. 2009a). Nineteen PFAAs were analyzed, and 12 were above the limit of quantification (LOQ; 0.05 ng/mL serum for all PFAAs reported in this study) in one or more samples. For quantification of PFOS, the total area of the linear and branched isomers was integrated. The quality of the analytical procedure was monitored by analyzing in-house quality control samples and human serum samples from interlaboratory comparison exercises (Institut national de santé publique Québec 2009). For the interlaboratory comparison exercises, the coefficient of variation for PFOA was between 3% and 9%, depending on the concentration of the reference sample. No traces of PFAAs above LOQ were observed in any of the procedural blanks.

*Offspring biomarkers of adiposity.* In short, adiponectin was quantified by a time-resolved immunofluorometric assay based on two antibodies and recombinant human adiponectin (R&D Systems, Abingdon, United Kingdom), as previously described (Frystyk et al. 2005). Leptin was determined by a time-resolved immunofluorometric assay based on commercial reagents (R&D Systems) using recombinant human leptin as the standard; otherwise, quantification was carried out essentially as for adiponectin (Frystyk et al. 2005). Plasma insulin concentrations were determined by the commercial Insulin ELISA kit (Dako, Copenhagen, Denmark).

*Outcome measures.* Body mass index (BMI) and waist circumference measures from the clinical examination were used when available (*n* = 423). Self-reported measures from the Web-based questionnaire were used for the remaining 242 subjects. Although subjects tend to underestimate their weight by self-report (Tokmakidis et al. 2007), relative ranks appeared to be retained, as the correlation between measured and self-reported BMI was 0.91 for both sexes (Spearman *r*, *n* = 423). Use of self-reported measures does, however, result in underestimation of cases when dichotomizing subjects as overweight and obese (BMI ≥ 25 kg/m^2^). In our data the sensitivity coefficient and specificity coefficients were 0.46 and 0.99, respectively, for identifying overweight and obese female offspring based on self-report. The corresponding numbers were 0.63 and 0.98 for male offspring, respectively.

*Statistical analyses.* The mean and SD were used to describe normal variables, whereas median and interquartile range (IQR) were used for skewed variables. Student *t*-tests or chi-square tests were used to test departures from the null hypothesis for normal outcomes, and the Kruskal–Wallis test was used for skewed outcomes. Linear regression was used for continuous outcomes. For dichotomous outcomes, relative risk (RR) was estimated using log-Poisson regression with robust variance estimation, as implemented in PROC GENMODE in SAS (version 9.2; SAS Institute Inc., Cary, NC, USA). All analyses were performed for males and females separately. When examining association with anthropometric outcomes, maternal PFOA concentrations were divided into quartiles and a test for linear trend was performed by using the ordinal values. For the biomarker analysis, PFOA was entered as a continuous variable.

When estimating the association between *in utero* PFOA concentration and offspring anthropometry at 20 years of age, we adjusted for the following potential confounders identified *a priori*: maternal age (continuous, no missing); maternal education (elementary schooling, high school or technical schooling, university education, higher academic education, other education, 6% missing); maternal smoking (never, < 10 and ≥ 10 cigarettes/day, 6% missing); pre-pregnancy BMI [restricted cubic spline regression (Durrleman and Simon 1989), 2% missing]; parity (0, 1, ≥ 2, no missing); infant birth weight (continuous, 0.2% missing); and offspring age at follow-up (continuous, 1% missing). A total of 67 subjects (10%) had missing values for one or more of the covariates included. Maternal age and parity were included because they are known predictors of PFAA exposure (Fei et al. 2007). Maternal education and smoking were included to account for potential social and lifestyle confounding. Pre-pregnancy BMI and birth weight were included because they are known predictors of offspring BMI (Reynolds et al. 2010). Missing covariate values were substituted using multiple imputation, as implemented in PROC MI in SAS (SAS Institute, Inc.).

Because of skewed distributions, offspring biomarkers of adiposity were log-transformed when examining their association with maternal PFOA concentrations, and estimates were adjusted for offspring age and timing of blood sample collection.

## Results

Non-significant differences in maternal PFOA concentration, prepregnancy BMI, and parity were observed between mothers whose offspring did not participate in follow-up, mothers whose offspring completed the Web-based questionnaire only, and mothers whose offspring participated in the clinical examination (Table 1). However, mothers whose offspring did not participate in follow-up were more likely to smoke during pregnancy. Females who participated in clinical examination had significantly lower BMI (≈ 0.8 kg/m^2^) compared with those who completed the Web-based questionnaire only, whereas non-significant differences were observed for waist circumference and current smoking. Male offspring who participated in the clinical examination were not significantly different from those who completed the Web-based questionnaire only with regard to BMI, waist circumference, and current smoking.

**Table 1:** Maternal and offspring characteristics at baseline and follow-up according to level of participation.

Characteristics	Offspring not in follow-up	Offspring only filling out Web-based questionnaire	Offspring participating in clinical examination	p-Value
Maternal		n = 209		n = 242		n = 423		
PFOA (ng/mL)a		3.7 (1.8)		3.7 (2.0)		3.7 (2.0)		0.68b
Prepregnancy BMI (kg/m2)		21.8 ± 3.8		21.1 ± 2.5		21.4 ± 3.0		0.06c
Nulliparous (%)		54		57		59		0.42d
Smoking during pregnancy (%)		49		36		37		0.01d
Male offspring at follow-up				n = 150		n = 170		
BMI (kg/m2)e				22.7 ± 2.9		22.6 ± 2.9		0.62c
Waist circumference (cm)e				85.5 ± 10.6		83.5 ± 8.9		0.08c
Current smoker (%)				19		18		0.75d
Female offspring at follow-up				n = 92		n = 253		
BMI (kg/m2)e				22.3 ± 3.2		21.5 ± 3.0		0.03c
Waist circumference (cm)e				79.8 ± 10.2		78.6 ± 9.7		0.32c
Current smoker (%)				21		17		0.45d
Based on the 874 mothers whose blood samples were analyzed for PFAAs in the Aarhus Birth Cohort (1988–1989). Values are median (IQR) for maternal PFOA concentration; otherwise, they are mean ± SD or %. aMaternal serum from gestational week 30. bKruskal–Wallis test of differences among participation groups. cF-test (type III) of differences among participation groups. dChi-square test of differences among participation groups. eComparison for offspring anthropometry is based on self-reported values.								

The median maternal PFOA serum concentration was 3.7 ng/mL (range: 0.1–19.8). Maternal PFOS, perfluorooctane sulfonamide (PFOSA), and perfluorononanoate (PFNA) concentrations increased across quartiles of PFOA concentration (Table 2). Eight other PFAAs were quantified but were not included in analyses because concentrations were low (median: ≤ 0.4 ng/mL) and were within a narrow range (IQR < 0.2 ng/mL). Mothers in the highest quartile of PFOA concentration were less likely to be parous (32% vs. 58%) and were younger (28.7 vs. 29.8 years) than mothers in the lowest quartile of PFOA when enrolled into the original study.

**Table 2:** Maternal characteristics during pregnancy among 665 participants from the Aarhus Birth Cohort (1988–1989).

Quartiles of maternal PFOA concentration			
Variables	Overall	1	2	3	4	p-Value
Serum concentration (ng/mL) a								
PFOA	3.7 (2.0)		2.4 (0.6)	3.3 (0.4)	4.2 (0.5)	5.8 (1.9)		
PFOS	21.5 (9.1)		16.0 (5.6)	20.2 (5.7)	23.6 (6.8)	28.5 1 (2.1)		< 0.0001b
PFOSA	1.1 (1.0)		0.7 (0.5)	1.1 (0.7)	1.3 (0.9)	1.5 (1.1)		< 0.0001b
PFNA	0.3 (0.2)		0.3 (0.2)	0.3 (0.2)	0.4 (0.2)	0.4 (0.3)		< 0.0001b
Physical characteristic								
Age (years)	29.2 ± 4.1		29.8 ± 3.8	29.2 ± 4.1	28.8 ± 3.9	28.7 ± 4.5		0.01c
Height (cm)	168 ± 6.0		167.4 ± 6.3	167.6 ± 6.5	167.8 ± 5.5	168.0 ± 5.7		0.43c
Prepregnancy BMI (kg/m2)	21.3 ± 2.9		21.1 ± 2.3	21.3 ± 3.2	21.2 ± 2.4	21.5 ± 3.2		0.25c
Birth weight	3.53 ± 0.52		3.62 ± 0.55	3.53 ± 0.57	3.49 ± 0.44	3.47 ± 0.52		0.009c
Smoking during pregnancy (%)	37		40	38	39	31		0.34d
Parous women (%)	42		58	39	38	32		< 0.0001d
Maternal education (%)								
Elementary schooling	10		13	12	7	8		0.0002d
High school or technical schooling	23		12	19	25	34		
University education	39		44	36	44	32		
Higher academic	18		25	20	14	14		
Other education or missing	10		6	13	11	12		
Values are median (IQR), mean ± SD, or %. aBlood (serum) samples were collected in gestational week 30; PFOA, PFNA, PFOS, and PFOSA were detected in all samples. bKruskal–Wallis test of differences across quartiles of maternal PFOA concentration. cStudent t-test of differences across quartiles of maternal PFOA concentration. dChi-square test of differences across quartiles of maternal PFOA concentration.								

The prevalence of overweight or obesity (BMI ≥ 25 kg/m^2^) was similar between male and female offspring (19% and 18%, respectively), but waist circumference above action level II (Lean et al. 1995) was less common in males (3.5% > 102 cm) than in females (16.2% > 88 cm) (Table 3). All males and 70% of females with waist circumference above action level II were overweight or obese. Females were more likely than males to report that they were currently on a diet (17% vs. 6%).

**Table 3:** Characteristics of male and female offspring at 20 years of age (2008–2009), Aarhus Birth Cohort (1988–1989).

Characteristic	Males	Females
Physical characteristic [mean ± SD or n (%)]		n = 320		n = 345
Age (years)		19.7 ± 0.4		19.9 ± 0.4
Waist circumference (cm)		84.3 ± 9.3		79.9 ± 9.4
BMI (kg/m2)		22.8 ± 2.9		22.2 ± 3.3
Waist circumference above action level IIa		11 (3.5)		56 (16.2)
Overweight or obeseb		60 (18.9)		61 (17.7)
Subjects in both a) and b)		11 (3.5)		39 (11.3)
Currently on a diet		20 (6.3)		60 (17.4)
Offspring attending clinical examination [median (IQR)]		n = 170		n = 252
Insulin (mmol/L)		37 (22)		42 (23)
Leptin (ng/L)		2.2 (3.1)		13.5 (12.6)
Adiponectin (μg/L)		6.8 (3.7)		9.4 (4.5)
Leptin/adiponectin ratio		0.3 (0.6)		1.5 (1.5)
aWaist circumference > 102 cm for males and > 88 cm for females (Lean et al. 1995). bBMI ≥ 25 kg/m2.				

Maternal PFOA concentrations were positively associated (*p* for trend < 0.05) with BMI and waist circumference among female offspring at 20 years of age (*n* = 345) in both unadjusted and covariate-adjusted analyses (Table 4). In adjusted analyses, female offspring whose mothers were in the highest quartile of PFOA concentration had 1.6 kg/m^2^ [95% confidence interval (CI): 0.6, 2.6] higher BMI and 4.3 cm (95% CI: 1.4, 7.3) higher waist circumference compared with offspring whose mothers were in the lowest quartile. No statistically significant associations were observed for males (*p* for trend > 0.05, *n* = 320). Female offspring of mothers in the highest versus lowest PFOA quartile were also more likely to be overweight [RR = 3.1 (95% CI: 1.4, 6.9)] and to have a waist circumference above action level II at 20 years of age [3.0 (95% CI: 1.3, 6.8)] (Table 5). PFOA was not associated with overweight in male offspring, and RR estimates were consistently null. Only 11 of 320 male offspring had a waist circumference above action level II; consequently, RR estimates were unstable with wide CIs.

**Table 4:** Associations^*a*^ between in utero exposure to PFOA and the offspring BMI and waist circumference at 20 years of age for females (n = 345) and males (n = 320).

PFOA in quartiles [median (range)]b		ΔBMI [mean (95% CI)]			ΔWaist circumference [mean (95% CI)]		
		Crude	Adjusted^c^	Crude	Adjusted^c^
Females									
1	2.3 (0.1–2.8)		Referent		Referent		Referent		Referent
2	3.2 (2.8–3.7)		0.2 (–0.7, 1.2)		0.4 (–0.6, 1.3)		1.0 (–1.7, 3.7)		1.4 (–1.4, 4.2)
3	4.2 (3.7–4.8)		0.8 (–0.2, 1.8)		0.9 (–0.1, 1.9)		0.9 (–1.9, 3.6)		1.2 (–1.7, 4.0)
4	5.8 (4.8–19.8)		1.6 (0.6, 2.5)		1.6 (0.6, 2.6)		4.2 (1.5, 6.9)		4.3 (1.4, 7.3)
p-Value for trendd			0.0007		0.001		0.005		0.006
Malese									
1	2.4 (1.2–2.8)		Referent		Referent		Referent		Referent
2	3.3 (2.8–3.7)		0.5 (–0.4, 1.4)		0.6 (–0.3, 1.5)		1.3 (–1.7, 4.3)		1.3 (–1.5, 4.1)
3	4.2 (3.7–4.8)		0.3 (–0.7, 1.2)		0.2 (–0.7, 1.1)		1.0 (–2.0, 4.0)		1.0 (–1.9, 3.8)
4	5.8 (4.8–16.6)		0.4 (–0.5, 1.3)		0.6 (–0.3, 1.5)		0.7 (–2.2, 3.6)		1.3 (–1.6, 4.1)
p-Value for trendd			0.47		0.30		0.72		0.48
aLinear regression with continuous outcome variables (BMI or waist circumference) and PFOA divided into quartiles. bNanograms per milliliter serum. cAdjusted for maternal age, maternal education, maternal preprepregnancy BMI, smoking during pregnancy, parity, infant birth weight, and offspring age at follow-up. dStudent’s t-test with PFOA included in the regression model as an ordinal variable. eWaist circumference was missing for two male offspring (n = 318).									

**Table 5:** Associations^*a*^ between in utero exposure to PFOA and risk of being overweight or having waist circumference above action level II at 20 years of age for females (n = 345) and males (n = 320).

PFOA in quartiles [median (range)]c		Overweight (BMI ≥ 25 kg/m2) [RR (95% CI)]			High waist circumferenceb [RR (95% CI)]		
		Cases/no.	Crude	Adjusted^d^	Cases/no.	Crude	Adjusted^d^
Females													
1	2.3 (0.1–2.8)		10/91		1.0		1.0		9/91		1.00		1.0
2	3.3 (2.8–3.7)		12/87		1.3 (0.6, 2.8)		1.5 (0.6, 3.5)		13/87		1.5 (0.7, 3.4)		1.7 (0.7, 4.1)
3	4.2 (3.7–4.8)		15/81		1.7 (0.8, 3.5)		2.0 (0.9, 4.7)		10/81		1.2 (0.5, 2.9)		1.3 (0.5, 3.2)
4	5.8 (4.8–19.8)		24/86		2.5 (1.3, 5.0)		3.1 (1.4, 6.9)		24/86		2.8 (1.4, 5.7)		3.0 (1.3, 6.8)
p-Value for trende					0.007		0.003				0.008		0.01
Malesf													
1	2.3 (1.2–2.8)		13/74		1.00		1.00		1/74		1.00		1.00
2	3.3 (2.8–3.7)		17/81		1.2 (0.6, 2.3)		1.2 (0.6, 2.6)		4/81		3.7 (0.4, 32.0)		6.1 (0.4, 90.0)
3	4.3 (3.7–4.8)		14/78		1.0 (0.5, 2.0)		1.0 (0.4, 2.2)		3/77		2.9 (0.3, 27.1)		7.3 (0.4, 121.4)
4	5.8 (4.8–16.6)		16/87		1.0 (0.5, 2.0)		1.1 (0.5, 2.6)		3/86		2.6 (0.3, 24.3)		13.3 (0.4, 298.9)
p-Value for trende					0.97		0.89				0.60		0.11
aLog-Poisson regression with robust variance estimation, as implemented in PROC GENMODE in SAS. bWaist circumference > 88 cm for females and > 102 cm for males (Lean et al. 1995). cNanograms per milliliter serum. dAdjusted for maternal age, maternal education, maternal preprepregnancy BMI, smoking during pregnancy, parity, infant birth weight, and offspring age at follow-up. eChi-square test (Type III) with PFOA included in the regression model as an ordinal variable. fWaist circumference was missing for two male offspring (n = 318).													

Among female participants who provided a blood sample at clinical examination (*n* = 252), maternal PFOA concentration was positively associated with insulin, leptin, and the leptin-adiponectin ratio and inversely associated with adiponectin levels (Table 6). The associations corresponded to a 2–7% change in these biomarkers per 1-unit increase in PFOA. Point estimates were similar for male offspring but non-significant (*n* = 170). Although the precision of the estimated associations between maternal PFOA and the biomarkers was reduced because of the small sample size for this analysis (particularly in males), associations between maternal PFOA and offspring anthropometry were comparable with those for the follow-up participants as a whole, supporting the validity of these estimates. For example, estimated increases in BMI comparing highest with lowest quartile of maternal PFOA concentration were 1.8 kg/m^2^ (95% CI: 0.6, 3.0) and 0.6 kg/m^2^ (95% CI: –0.7, 1.9) for female and male offspring who provided a blood sample at clinical examination, compared with 1.6 kg/m^2^ (95% CI: 0.6, 2.6) and 0.6 kg/m^2^ (95% CI: –0.3, 1.5) for all female and male participants, respectively.

**Table 6:** Association^*a*^ between in utero exposure to PFOA and offspring serum biomarkers of adiposity at 20 years of age.

Females (n = 252)					Males (n = 170)				
Biomarker	Percent changeb,c	95% CI	p-Value	Percent changec	95% CI	p-Value
Insulin		4.5		1.8, 7.2		0.001		2.2		–0.8, 5.3		0.15
Leptin		4.8		0.5, 9.4		0.03		4.5		–2.6, 12.1		0.21
Adiponectin		–2.3		–4.5, –0.2		0.03		–1.7		–4.6, 1.2		0.25
Leptin/adiponectin ratio		7.2		2.2, 12.5		0.004		6.3		–1.5, 14.6		0.11
The associations are limited to the subgroup of participants (n = 422) that provided blood samples at clinical examination. aLinear regression using log-transformation for the dependent variable (continuous). The results are adjusted for time of blood sample collection (e.g., 08:00–12:30) and offspring age at follow-up. bBlood sample was missing for one female subject who attended clinical examination. cPercent change in the outcome measure for 1-ng/mL change in serum PFOA.												

Concerning other PFAAs, we observed that PFOS, PFNA, and PFOSA (continuous variables) were in univariate analysis either borderline (*p* for trend = 0.05 for PFOSA) or significantly (*p* for trend < 0.05 for PFNA, PFOS) positively associated with female offspring BMI at 20 years. However, after adjusting for PFOA, the regression coefficients became non-significant (*p* for trend ≥ 0.56 in all cases, data not shown). We also found no evidence that the association between maternal PFOA concentration and offspring anthropometry was influenced by the presence of the other PFAAs quantified. For example, the estimated increase in female offspring BMI with a 1-ng/mL increase in maternal PFOA in a univariate analysis was 0.43 kg/m^2^ (95% CI: 0.25, 0.60) compared with 0.46 kg/m^2^ (95% CI: 0.21, 0.71) based on a model that included maternal PFOS, PFOSA, and PFNA as continuous terms.

Associations with offspring BMI were also stable with respect to maternal parity. For female offspring of nulliparous mothers (*n* = 191), we estimated a 0.46-kg/m^2^ (95% CI: 0.27, 0.66) increase in BMI per 1-ng/mL increase in PFOA, compared with 0.36 kg/m^2^ (95% CI: 0.03, 0.69) for female offspring of parous mothers (interaction *p*-value 0.65).

## Discussion

In this cohort of environmentally exposed pregnant women, we observed a positive association between *in utero* exposure to PFOA and the prevalence of overweight and a high waist circumference among female offspring, but not male offspring, at 20 years of age. In addition, *in utero* exposure to PFOA was associated with biomarkers of adiposity among male and female offspring, although estimates for males were not significant.

Animal studies have shown that early--life exposures to xenoestrogens such as diethylstilbestrol (Newbold et al. 2008) and bisphenol-A (Somm et al. 2009) can lead to permanent disruption in endocrine functions that regulate adiposity. One observation from these studies is that low-dose exposures may result in significant weight changes in the exposed animals only after adult age is reached, whereas a pattern of reduced weight in early infancy followed by rapid catch-up growth is often observed at higher doses (Newbold et al. 2008). Human studies have provided some support for these findings, including reports of rapid weight gain up to 3 years of age after *in utero* exposures to polychlorinated biphenyls (Verhulst et al. 2009) as well as increased overweight at 6 years of age after *in utero* exposure to hexachloro-benzene (Smink et al. 2008). However, metabolic pathways that might explain these findings are not well characterized, and long-term follow-up studies have not been conducted.

Indications that environmental exposures to PFAAs may affect cardiometabolic risk factors have, so far, been obtained from cross-sectional studies focusing on end points such as blood lipids (Nelson et al. 2010) and glucose homeostasis (Lin et al. 2009), but in these studies, findings with respect to anthro-pometry have been inconsistent. Interpretation of cross-sectional findings on anthropometry is complex, however, as absorption and distribution of PFAAs depend on related factors such as lean body mass and blood volume (Ahrens et al. 2009; Fei et al. 2007).

However, our findings are consistent with a recent experimental study on CD-1 mice that reported increased body weight in postpubertal female offspring after low-dose (0.01–0.3 mg/kg body weight) *in utero* exposure to PFOA (Hines et al. 2009). In that study, increases in weight were most pronounced in mid-life and were accompanied by elevated levels of leptin and insulin in the exposed animals. At higher doses (≥ 1mg/kg), weight gain was not observed. Furthermore, exposing the offspring after weaning as opposed to *in utero* also had no effect on weight gain. In at least two animal studies, researchers did not observe increased body weight after *in utero* exposure to PFOA (Butenhoff et al. 2004; Macon et al. 2011), but one study used higher doses (≥ 1mg/kg) (Butenhoff et al. 2004) and the other followed animals only until 12 weeks of age (Macon et al. 2011).

Similar to the findings of Hines et al. (2009), we observed significant associations between maternal PFOA and insulin and leptin in addition to adiponectin levels among female offspring. Changes in these biomarkers were modest, however, and corresponded to what could be expected from the observed increase in offspring BMI. The results from our biomarker analysis provide valuable insight, as elevated insulin levels predict central fat in both normal weight and overweight women (Carey et al. 1996). Therefore, these results provide added weight to our findings, as BMI and waist circumference are relatively crude measures of body composition.

The absence of an association between *in utero* PFOA exposure and weight gain among male offspring is noteworthy but not unexpected. Sex-specific differences with respect to weight gain have been observed for xenoestrogens, where female offspring appear to be more affected (Newbold et al. 2008; Somm et al. 2009). Results for male offspring were not reported in the CD-1 mouse study (Hines et al. 2009), and experimental studies have suggested that reproductive development, but not weight gain, may be a more sensitive end point for males with respect to developmental exposures to PFAAs (Shi et al. 2007). Despite these observations, results for male offspring in our study should be interpreted cautiously, as many study participants were still in their late adolescence, and relatively few males had high waist circumference compared with females. Because girls gain more fat mass and boys gain more fat-free mass during adolescent age (Ahmed et al. 1999), weight gain in male offspring at a later age when accumulation of fat mass increases cannot be excluded.

Concerning mechanism, there are at least three potential pathways by which *in utero* exposure to PFOA might affect offspring weight. First, PFOA may interfere with ovary development *in utero*, leading to impaired estrogen synthesis among female offspring. This mechanism is supported by the observation that *in utero* PFOA exposure did not affect weight gain in the previously mentioned study on CD-1 mice when ovariectomy was performed in exposed offspring after delivery (Hines et al. 2009). Second, PFAAs may interact with the peroxisome proliferator--activated receptors (PPAR) PPARα and PPARγ, which are involved in lipid metabolism in adipocytes (Hines et al. 2009). Whether such an interaction can lead to sex-specific differences in weight gain remains unclear. Third, thyroid hormones might play some role: The results of one cross-sectional study suggested that PFOA exposure may lead to an increased risk of thyroid disease (Melzer et al. 2010), which is more common in females than males. A recent study from the C8 Health project also observed a modest positive association of PFOA with serum thyroxine and an inverse association with T3 uptake (Knox et al. 2011). The overall evidence linking PFAAs to thyroid function is still weak (Steenland et al. 2010), particularly as prospective studies have not been conducted, and no clear effect on thyroid function has been observed in occupationally exposed individuals (Olsen and Zobel 2007).

A unique strength of our study is the long-term prospective follow-up with outcome assessment at an age where weight changes due to linear growth have leveled off, and it was possible to adjust for several prenatal factors including maternal BMI, smoking, and education. We were also able to support our findings on offspring anthropometry with assessment on serum biomarkers of adiposity in a subset of study participants. Furthermore, quantification of a total of 12 PFAAs made it possible to conclude that PFAAs other than PFOA were unlikely to play an important role in our study population, either because of a low concentration or a lack of association after adjusting for PFOA.

Concerning limitations, we acknowledge that uncertainties regarding routes of human exposure to PFAAs may have resulted in a failure to identify important confounders. We also acknowledge that maternal PFOA concentrations in late gestation may be influenced by factors such as blood volume expansion, decreased albumin concentration, and fetal uptake (Fei et al. 2007; Frederiksen 2001), and these factors may predict fetal outcomes such as birth weight (Savitz 2007). However, with a temporal separation of 20 years, our findings should be less prone to such influence. Furthermore, PFOA concentrations in samples collected at around gestational weeks 6–12 and 24 (Olsen et al. 2001) and in cord blood have been observed to be highly cor-related (*r* ≈ 0.8–0.9) (Fei et al. 2007).

Finally, as with all longitudinal studies, losses during follow-up are inevitable, and this is perhaps the main limitation of our study. Having quantified maternal PFOA concentrations in 874 (or 72%) of 1,212 women who were originally invited to participate, it is reassuring that exposure levels were non-differential with respect to level of participation in the -follow-up study. A higher prevalence of smoking among mothers whose offspring did not participate suggests that losses due to follow-up were related to some extent to maternal lifestyle. There were relatively modest differences in anthropometry between offspring who participated in the clinical examination and those who completed the Web-based questionnaire only. Nonetheless, our secondary analyses of offspring biomarkers of adiposity may be compromised because of the low participation rate, particularly among males.

## Conclusion

In a cohort of environmentally exposed pregnant women, a positive association between *in utero* exposure to PFOA and the prevalence of overweight and high waist circumference was observed in female offspring at 20 years of age. Our findings are in line with recent experimental findings (Hines et al. 2009) and provide added support for the hypothesis that early-life exposure to certain endocrine disruptors, even at low concentrations, may play a role in the current obesity epidemic. Given the widespread detection of PFOA in humans and wildlife and observed increase in related perfluorinated compounds of similar biological potential (Harada et al. 2011; Haug et al. 2009b), it is of considerable public health importance to understand and eliminate pathways of human exposures to PFAAs.
